# Simultaneous Head and Neck and Lung Cancers: Implications and Therapeutic Management Timing

**DOI:** 10.1111/coa.70066

**Published:** 2025-12-07

**Authors:** Dario Ebode, France Truong, Caroline Halimi, Axelle Dupont, Valerie Gounant, Sandrine Faivre, Muriel Hourseau, Béatrix Barry, Esteban Brenet, Diane Evrard

**Affiliations:** ^1^ Department of Oto‐Rhino‐Laryngology Head and Neck Surgery APHM, La Conception University Hospital, Aix‐Marseille University Marseille France; ^2^ Department of Otorhinolaryngology Reims University Hospital Reims France; ^3^ Department of Otorhinolaryngology Bichat University Hospital, University of Paris Paris France; ^4^ Clinical Research, Biostatistics and Epidemiology Department AP‐HP Nord‐Université de Paris, HUPNVS Paris Cedex France; ^5^ INSERM CIC‐EC 1425, Hôpital Bichat Claude Bernard Paris France; ^6^ Department of Thoracic Oncology Bichat University Hospital, University of Paris Paris France; ^7^ Medical Oncology Department Saint‐Louis Hospital, University of Paris Paris France; ^8^ Pathology Department Bichat University Hospital, University of Paris Paris France

**Keywords:** head and neck cancer, lung cancer, second cancer, squamous cell carcinoma, treatment delay

## Abstract

**Objectives:**

This study aims to describe the characteristics of patients with simultaneous head and neck squamous cell carcinomas (HNSCCs) and lung cancer and to assess the impact of diagnosis‐to‐treatment interval (DTI) and treatment choices on outcomes.

**Design and Setting:**

A bicentric retrospective observational study conducted between 2003 and 2020 in two tertiary referral academic hospitals.

**Participants:**

Forty‐three patients diagnosed with both HNSCC and lung cancer within 1 month were compared to a 1:2 control group of patients diagnosed with HNSCC alone. The groups were matched by tumour location, TNM stage and treatment centre. Multivariate analysis was used to assess factors influencing DTI, and survival analysis was conducted using the Kaplan–Meier estimator.

**Main Outcome Measures:**

Primary outcomes included DTI and overall survival.

**Results:**

Patients with simultaneous cancers were predominantly male (84%) with heavy smoking histories. Most HNSCC cases (84%) were diagnosed at advanced stages (III/IV), while lung cancers were predominantly early stage (I–II). Patients with simultaneous cancers had significantly longer DTIs (median 43 days, IQR [32.8–85.8]) compared to controls (28 days, IQR [19.0–38.0]) (*p* < 0.001; HR = 0.35, *p* = 0.0001). As expected, overall survival was markedly worse for patients with simultaneous cancers (median 17.8 months, 95% CI [12.6–31.0]) versus controls (56.5 months, 95% CI [38.4–], *p* < 0.001).

**Conclusion:**

Prolonged DTI may contribute to the poorer survival outcomes in patients with simultaneous cancers. Strategies to reduce delays, such as performing concurrent bronchoscopy and endoscopy, should be explored.

## Key Points

- Simultaneous diagnoses of head and neck squamous cell carcinomas (HNSCCs) and lung cancer occur in 0.6%–1.4% of HNSCC patients, predominantly affecting males with heavy smoking histories.
- Among patients with simultaneous cancers, HNSCC cases were frequently advanced stage (III/IV), while lung cancers were primarily early‐stage adenocarcinomas (I–II in 60% of cases).
- Patients with simultaneous cancers experienced significantly longer diagnosis‐to‐treatment interval (DTIs) (median 43 days) compared to HNSCC‐only patients (28 days), with delays persisting even after adjusting for confounders (HR = 0.35, *p* = 0.0001).
- Simultaneous cancer patients had significantly worse overall survival (median 17.8 months) and recurrence‐free survival (median 31.5 months) compared to controls, highlighting the clinical impact of diagnostic and treatment delays.
- This study emphasises the need to reduce DTIs in simultaneous cancer cases, potentially through integrated diagnostic procedures like concurrent bronchoscopy and endoscopy, aligning with guidelines recommending treatment initiation within 28 days.

## 1 Introduction

Head and neck squamous cell carcinomas (HNSCCs) and lung cancers rank as the eighth and second most prevalent cancers worldwide, respectively [1]. Due to their significant link to tobacco use, between 0.6% and 1.4% of patients with HNSCC are also diagnosed with concurrent lung cancer [2, 3].

Simultaneous cancers (SCs) are defined as two primary cancers diagnosed within 1 month of each other [4]. They fall under the category of synchronous cancers (diagnosed less than 6 months apart) and differ from metachronous cancers (diagnosed more than 6 months apart). SCs require special management because both tumours need to be evaluated at the same time to distinguish between metastatic cancer and two separate primary cancers. This is particularly important for cases of simultaneous HNSCC and lung cancer, as the lungs are a common site for metastasis of HNSCC. As SCs remain rare, there is limited information available about this patient population in the literature.

Coordinating check‐ups and treatment plans can pose significant challenges and time constraints. Given the location of HNSCC, the aerodigestive tract can become rapidly obstructed, making the time before treatment initiation critical. This period can be broken down into several phases. The first phase is the ‘patient delay’, which goes from the first symptoms to the first medical appointment. Once the consultation occurs, the ‘professional delay’ begins, lasting until the initiation of treatment. The professional delay can further be divided into the ‘delay to biopsy’ and the ‘diagnosis‐to‐treatment interval’ (DTI). Multiple factors impacting the DTI have been identified, such as the tumour's stage, anatomic location, the type of treatment received, as well as the performance status [5, 6, 7].

The primary objective of this study is to describe the characteristics of patients with simultaneous HNSCC and lung cancer, and to measure their DTI.

## 2 Methods

Authorisation to conduct this study was obtained. This study was conducted using fully anonymised retrospective data. According to institutional guidelines, written informed consent was not required. Ethical approval was obtained from the local Ethical Committee (No‐IRB 00006477), which confirmed the exemption from informed consent. STROBE guidelines were followed.

Patients from two tertiary referral centres in France were included. All consecutive patients diagnosed with lung cancer and HNNSC within 1 month between 2003 and 2020 were included in this observational retrospective study. In cases of doubt regarding the primary nature of a lung cancer, the multidisciplinary tumour board's conclusion was considered as the reference to distinguish a primary from a secondary lung tumour.

A control group of patients with HNSCC alone was formed in a 1:2 ratio. The two groups were paired according to tumour location, TNM stage and hospital of treatment.

Exclusion criteria were the absence of histologic evidence of each tumour and the metastatic stage of HNSCC.

The clinical characteristics of each patient, the characteristics of the tumour and the chronology of their management were gathered. The ‘patient's delay’ was defined as the delay between the beginning of the symptoms and the first appointment with a specialist. The ‘DTI’ was defined as the delay between the biopsy of the tumour and the initiation of the treatment. Malnutrition was defined as a loss of > 10% of the body weight. Overall survival was defined as the time from the date of biopsy to the date of death or censoring if applicable. Recurrence‐free survival was defined as the time from the date of first follow‐up to the date of death or recurrence (first event) or censoring, if applicable.

Categorical variables were compared between groups using the chi‐square test or the Fisher's exact test when applicable. Quantitative variables were compared by the Wilcoxon test. Survival curves were estimated with the Kaplan–Meier estimator. Comparisons between groups were performed using the log rank test. Univariate analyses of factors associated with the DTI were performed using Cox models. Variables with *p* < 0.20 were included in the final multivariate model. Hazard ratios (HRs) with 95% CIs are given. The statistical significance threshold was set at *p* < 0.05. All analyses were performed by a dedicated statistician who used R 4.4.

## 3 Results

### 3.1 Population

Forty‐three patients with HNSCC and simultaneous lung cancer at the time of diagnosis between 2003 and 2020 were included. They were paired with a control group of 86 patients, for a total of 129 patients. This represents approximately 0.8% of each newly diagnosed HNSCC in our two tertiary referral centres.

Patients' characteristics are summarised in Table 1.

**TABLE 1:** Characteristics of the population.

Characteristic	Simultaneous group (*n* = 43)	Control group (*n* = 86)	*p*
Age at time of diagnosis	65 [58; 68]	63 [58; 68]	0.68
Median [IQR]			
Sex (*n*, %)			0.53
Male	36 (84%)	68 (79%)	
Female	7 (16%)	18 (21%)	
Tobacco consumption (*n*, %)	43 (100%)	76 (88%)	**0.03**
Cumulative tobacco consumption (PY, median)	60	40	**< 0.001**
Alcohol consumption (*n*, %)	29 (67%)	50 (58%)	0.31
ECOG performance status (*n*, %)			**< 0.01**
0	19 (44%)	60 (70%)	
1	19 (44%)	19 (22%)	
2	3 (7%)	7 (8.1%)	
3	2 (4.7%)	0	
4	0	0	
Immunosuppression (*n*, %)	2 (4.7%)	4 (4.7%)	1.00
Malnutrition (*n*, %)	20 (47%)	25 (29%)	0.05
History of cancer (*n*, %)	8 (19%)	10 (12%)	0.28
Otolaryngological symptoms (*n*, %)			0.3
None	4 (9.3%)	4 (4.8%)	
Odynophagia	7 (16%)	16 (19%)	
Dysphagia	14 (33%)	20 (24%)	
Dysphonia	10 (23%)	13 (15%)	
Otalgia	1 (2.3%)	2 (2.4%)	
Tumour/ulceration	4 (9.3%)	8 (9.5%)	
Adenopathy	0	5 (6%)	
Dyspnoea	3 (7%)	16 (19%)	
Location (*n*, %)			1.00
Oral cavity	6 (14%)	12 (14%)	
Oropharynx	12 (28%)	24 (28%)	
Hypopharynx	9 (21%)	18 (21%)	
Larynx	16 (37%)	32 (37%)	
TNM staging (*n*, %)			
T Stage			1.0
1	4 (9.3%)	8	
2	11 (26%)	22	
3	15 (35%)	30	
4	13 (30%)	26	
N Stage			1.00
0	21 (49%)	42	
1	8 (19%)	16	
2	14 (33%)	28	
M Stage			1.00
0	43 (100%)	86	
1	0	0	
p16+ status (*n*, %)	1/38 (2.3%)	8/36 (9.3%)	**0.013**
Curative intent treatment (*n*, %)	39 (91%)	84 (98%)	0.095
Main treatment received (*n*, %)			0.22
None	1 (2.3%)	2 (2.3%)	
Surgery	22 (51%)	48 (56%)	
Radiotherapy	2 (4.7%)	8 (9.3%)	
Radiochemotherapy	14 (33%)	27 (31%)	
Chemotherapy	4 (9.3%)	1 (1.2%)	

*Note*: The bold value indicates *p* values < 0.05.

Characteristics of lung cancers are found in Table 2. Notably, 60% of them were found at an early stage (I–II). Thirty‐five patients (81%) did not report any symptoms related to their lung cancer. Diagnosis was mainly obtained by transthoracic needle biopsy (42%) and by flexible bronchoscopy in 27% of the cases. Most of the cases were adenocarcinoma (44%).

**TABLE 2:** Characteristics of the lung cancers.

Characteristic	*N* (%)
Stage (lungs)	
I	17 (44%)
II	6 (15%)
III	7 (18%)
IV	9 (23.3%)
Histology	
NSCLC	36 (84%)
Adenocarcinoma	20 (47%)
SCC	14 (33%)
Unclassified	2 (5%)
SCLC	6 (14%)
Other	1 (2%)
First line of treatment	
Surgery	19 (46%)
Chemotherapy	8 (20%)
Radiotherapy	4 (10%)
Radiochemotherapy	8 (20%)
None	2 (5%)

Abbreviations: *N*, number of patients; NSCLC, non–small cell lung cancer; SCLC, small cell lung cancer.

Patients with SCs differed from the control group in three key characteristics:

– Performance status was worse with a majority of patients (70%) graded 0 in the control group (*p* = 0.01)
– The cumulative consumption of tobacco was significantly lower in the control group (40 vs. 60 pack‐years [PY]) (*p* < 0.01)
– A p16‐positive status was more frequent in the control group (5% vs. 14%, *p* = 0.002). However, among p16‐positive patients in the control group, five of eight had a smoking history > 20 PYs.

### 3.2 Treatment of the HNSCC

#### 3.2.1 Treatment Received

Surgical treatment was the most common therapeutic approach, accounting for 51% of cases, followed by concomitant radiochemotherapy at 33%. No significant difference in treatment choice was observed between the SC group and the control group, with both groups showing similar proportions of patients treated with surgery and radiotherapy. In addition, four patients with SC (9%) received palliative treatment as their first‐line therapy and one patient (5%) refused any treatment. This was comparable to the control group, where three patients received palliative treatment and two patients refused treatment (*p* = 0.095).

### 3.3 Analysis of the Delays

DTI was significantly higher for patients with SC, with a median of 43 versus 28 days in the control group (*p* ≤ 0.001). This difference persisted in a multivariate analysis after adjusting for potential confusing factors (HR = 0.35, *p* = 0.0001).

The only other identified factor influencing the DTI after a multivariate analysis was a PS grade 2–3, which was associated with a longer DTI (HR = 3.44, *p* = 0.0004) (Table 3).

**TABLE 3:** Multivariate analysis of the DTI.

Characteristic	HR	CI	*p*
Simultaneous vs. Control	0.33	[0.21–0.51]	**< 0.0001**
Dysphonia	0.73	[0.44–1.21]	0.23
Surgical treatment	1.69	[1.13–2.52]	0.011
ECOG PS 1 vs. 0	0.94	[0.61–1.44]	0.76
ECOG PS 2–3 vs. 0	4.03	[2–8.15]	**0.0001**

*Note*: The bold value indicates *p* values < 0.05.

Abbreviations: CI, confidence interval; HR, hazard ratio; PS, performance status; PY, pack‐year.

The delay between the first appointment and biopsy of the HNSCC was similar in both groups. Similarly, no significant difference was observed in patient delay between the two groups.

### 3.4 Survival

**FIGURE 1:**
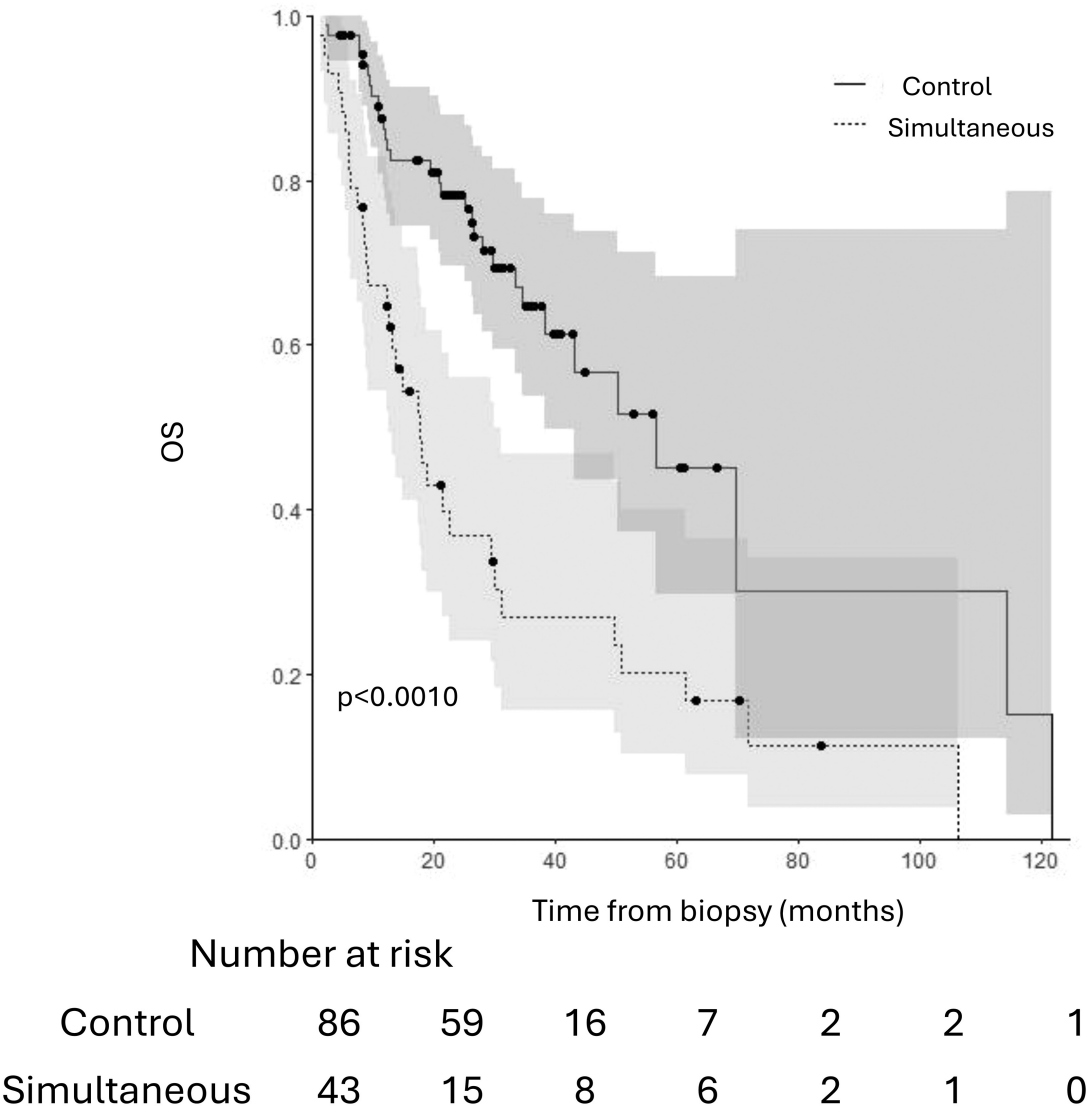
Overall survival in ‘simultaneous’ and ‘control’ group.

The median follow‐up was 26 months in the simultaneous group versus 30 months in the control group (*p* = 0.39). Overall survival was significantly lower in the synchronous group with a median survival of 17.8 months [12.6–31.0] compared to 56.5 months [38.4–] in the control group (*p* < 0.001) (Figure 1).

**FIGURE 2:**
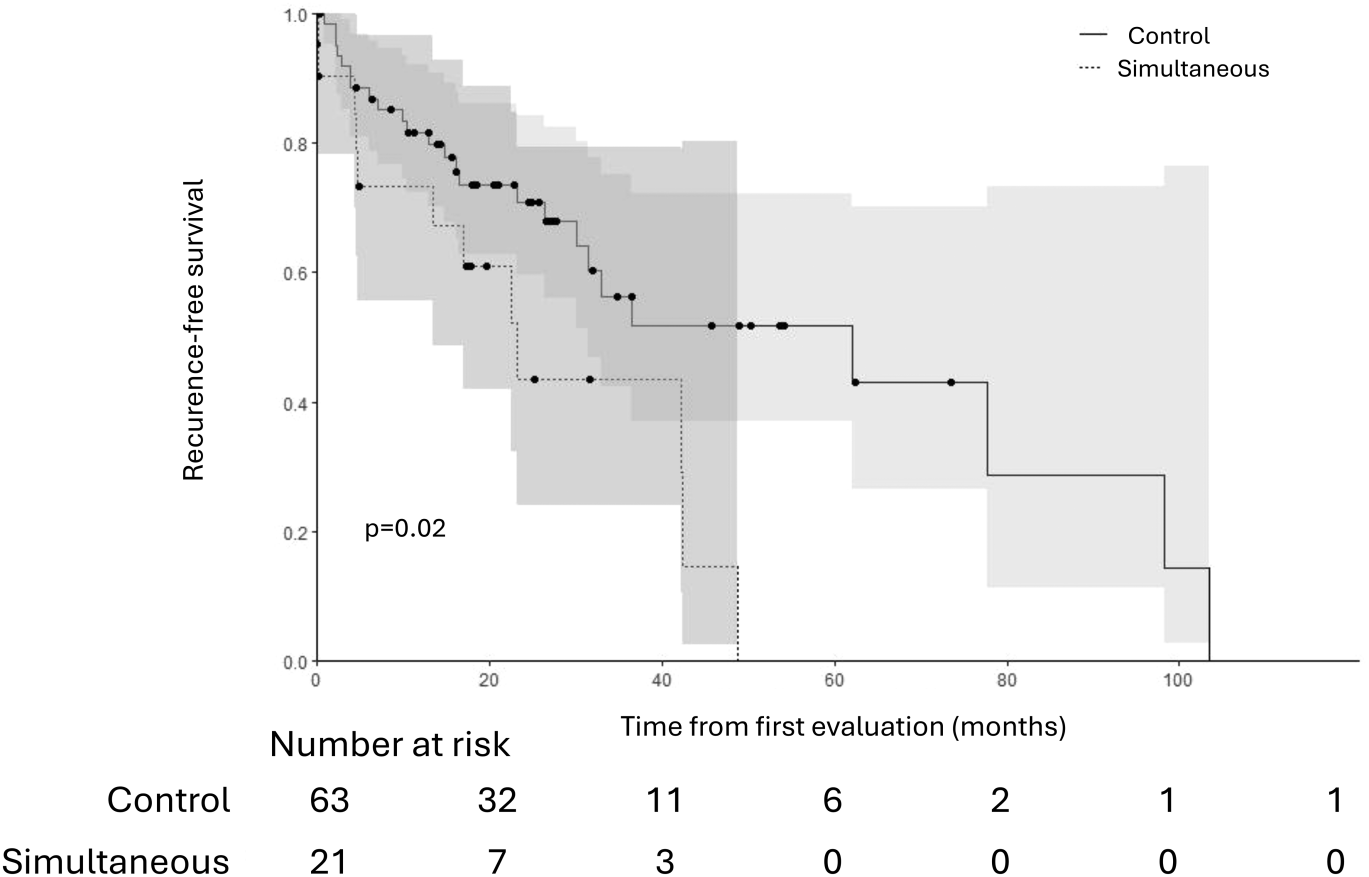
Recurrence‐free survival in ‘simultaneous’ and ‘control’ group.

Recurrence‐free survival was also significantly inferior in the simultaneous group (31.5 months [10.3–] vs. 36.5 months [30.1–]) (*p* = 0.02) (Table 4; Figure 2).

**TABLE 4:** Overall survival, recurrence‐free survival and remission rate in both groups.

	Simultaneous group	Control group	*p*
Remission of the HNSCC (*n*, %)	21 (49%)	65 (76%)	**0.005**
Recurrence‐free survival in months (median [95% CI])	31.5 [10.3–]	36.5 [30.1–]	**0.02**
Overall survival in months (median [95% CI])	17.8 [12.6–31.0]	56.5 [38.4–]	**< 0.001**
Overall survival of patients with curative intent treatment (median [95% CI])	18.9 [13.5–49.7]	56.5 [43.1–]	**< 0.001**

*Note*: The bold value indicates *p* values < 0.05.

Abbreviations: HNSCC, head and neck squamous cell carcinoma; SCC, squamous cell carcinoma.

## 4 Discussion

This study is the first to analyse medical care delay in this population. Only a few studies have specifically examined synchronous HNSCC and lung cancer [2, 4, 8, 9, 10]. Among them, only one has focused on SCs.

### 4.1 Population

As expected, patients with SC were all heavy smokers with a cumulative tobacco consumption significantly higher than in patients with isolated HNSCC. The proportion of smokers is also superior to what is found in large epidemiologic studies on isolated HNSCC, generally between 70% and 90% [11]. This finding is in tune with other studies, which also reported a high proportion of smokers in patients with synchronous cancers [2, 4, 8, 9, 10]. The cumulative consumption was not always reported in these studies, but Tamjid et al. observed a consumption that was similar to ours, reaching 50 PY [9]. Most of the HNSCC were located in the larynx, which was expected given that tobacco is the main risk factor for both laryngeal and lung cancer. Previous studies have already highlighted the high prevalence of laryngeal cancers in this population, but the distribution for the other locations varies [2, 4, 8, 9, 10].

Although metastatic HNSCC was excluded from our cohort to prevent any confusion, 84% of the HNSCC cases were diagnosed at advanced stages (Stage III/IV). This proportion is higher than anticipated, as a recent large epidemiologic study on isolated HNSCC reported 73% of advanced‐stage cancers [7]. This predominance of advanced‐stage cancers is consistent with the data on synchronous tumours [8, 9].

Regarding lung cancers, most of them were adenocarcinoma, and only 19% of them were SCC. This is consistent with the French distribution of lung cancer at that time [12]. Similar studies usually reported more SCC than adenocarcinoma [2, 4, 8, 9]. However, there is a great variability in the adenocarcinoma/SCC ratio, depending on the geographic location and the time period [12, 13, 14]. Thus, the study from Tamjid et al., which is the most recent on synchronous HNSCC and lung cancers, also describes similar results. However, we cannot exclude that some primary lung SCC might have been considered as metastasis from the HNSCC at the time of diagnosis and were therefore excluded from our study. As there are no histological characteristics to identify a HNSCC metastasis from a primary SCC lung cancer, one usually relies on clinical or radiological findings [15, 16]. Recently, some authors have shown that the next‐generation sequencing (NGS) technology may help in this matter. Two tumours having the same genetic profile have a high probability of having the same origin [17, 18].

Lung cancers were found at early stages (I and II) in 60% of cases, whereas they represent only 25% of lung cancers diagnosed in the standard population [12]. It is mainly explained by the incidental discovery of most of these cancers before symptoms appear. This finding highlights the relevance of lung cancer screening in all patients with HNSCC.

### 4.2 Survival

In our cohort, the median overall survival of patients with simultaneous tumours was 17.8 months [12.6–31.0]. In a large prospective study regarding HNSCC, Rennemo et al. reported a median overall survival of 45 months [19]. Therefore, the presence of simultaneous lung tumours appears to be a poor prognostic factor. The comparison with the control group confirmed this result, with a significantly lower survival compared to our control group (17.8 months [12.6–31.0] vs. 56.5 months, *p* < 0.001).

Compared to other studies on simultaneous tumours, the overall survival in our study is similar to the report by Griffioen et al. (19 months) but significantly lower than the rate reported by Kuriakose et al., who found a 5‐year survival rate of 47% (median survival close to 60 months) [4, 8]. However, the latter study only included patients who had received curative intent treatment.

### 4.3 Delay in Treatment Initiation

The DTI for patients with SC was significantly longer than that of the control group (43 vs. 28 days, *p* < 0.001). This difference was confirmed in a multivariate analysis, which demonstrated that simultaneous lung cancer is an independent factor prolonging the DTI. The DTI in our control group aligns with the findings of Guizard et al. in a large multicentric study of HNSCC in France (35 days [21–54]) [7].

An extended DTI is associated with a decrease in tumour control after radiotherapy and an increased risk of cancer recurrence [20]. Its impact on mortality is more controversial, but a recent meta‐analysis reported 9 of 13 studies showing that an increased DTI had a negative impact on overall survival [21]. The psychological effects on the patients and their relatives are not to be undermined, either, with increased stress and anxiety during the delay [22]. The main reason for this prolonged DTI seems to be an increased workup delay. As it is necessary to differentiate a simultaneous lung cancer from a pulmonary metastasis, ENT surgeons often need to wait until a complete pulmonary workup is done to choose the best treatment for the patient.

### 4.4 Improving the Delays

For patients with SCs, the main factor contributing to reduced survival remains the presence of a second primary malignancy, as demonstrated in multiple studies on synchronous cancers [2, 8, 9, 10]. Having two concurrent cancers increases the risk of tumour progression, metastasis and recurrence. Managing them also carries a higher morbidity risk. Given the poorer survival outcomes in this patient group, particular attention should be given to minimising the DTI to optimise prognosis.

The French ENT Society (SFORL) recommends a professional delay of 2 weeks (including the consultation–biopsy delay plus the DTI), and inferior to 28 days. In the simultaneous group, the DTI alone was longer than 28 days in 88% of cases. Thus, it appears that strategies should be found to decrease the workup time of these patients.

An interesting solution could be to perform flexible bronchoscopy (or endobronchial ultrasound [EBUS]) and the panendoscopy at the same time in case of strong suspicion of synchronous lung cancer. This method could potentially decrease the DTI by approximately 15 days (the estimated average time to schedule these examinations and for histological analysis). Performing bronchoscopy under general anaesthesia would also be more comfortable for the patient.

Later on, multidisciplinary tumour boards including ENT surgeons and thoracic oncologists are required to ensure optimal management and create a common schedule for treatment and follow‐up.

### 4.5 Strategy for Treatment

Regarding the sequencing of the treatment, we find in our study a similar pattern to the one described by Kuriakose et al. [4]. If one of the cancers was treated by surgery and the other by radiotherapy, the surgical treatment was performed first. If both cancers were treated by surgery, the HNSCC was treated first. This strategy is based on a longer recovery time after lung surgery than after HNSCC surgery [4]. Moreover, lung surgery has an impact on the patient's pulmonary capacity and could complicate the HNSCC surgery.

Furthermore, prioritising the treatment of lung cancer over HNSCC could risk the obstruction of the respiratory tract. Unlike lung cancer, which can remain asymptomatic for an extended period, HNSCC can rapidly cause respiratory distress and malnutrition. However, in the case of aggressive lung cancers such as small cell lung cancer (SCLC), this treatment protocol should be carefully evaluated.

In the future, immunotherapies with PD‐1/PD‐L1 may play a greater role in the treatment of patients with SCs, as this therapeutic class could have a parallel action on both tumours. However, it seems that this treatment does not show the same efficacy depending on the type of tumour. Some patients, therefore, show dissociated responses to each cancer. Several clinical cases of synchronous tumours, including a lung lesion, have been published with mixed outcomes [23, 24, 25].

### 4.6 Limits

The low prevalence of synchronous cancers in the general population required us to collect data retrospectively. However, this data was gathered over a 17‐year period, during which both the patient population and best practice guidelines for diagnosing HNSCC and lung cancer may have changed. For example, the use of EBUS has only become widespread in the past decade. In addition, the study included a small number of patients, which sometimes lacked the power for subgroup analysis to provide accurate results, particularly concerning recurrence‐free survival. Furthermore, there is likely a recruitment bias, as some synchronous primary lung cancers may have been excluded if they were considered HNSCC metastases at diagnosis. In the future, systematic analysis of the biomolecular characteristics of the tumours could help limit this bias.

## 5 Conclusion

The presence of a second cancer contributes to delays in the initiation of treatment in patients with HNSCC and may negatively impact overall survival.

Improved coordination is essential to minimise these delays and enhance treatment outcomes for this patient population.

## Author Contributions

**Dario Ebode:** writing, data collection. **France Truong:** data collection. **Caroline Halimi:** reviewing. **Axelle Dupont:** statistical analysis. **Valerie Gounant:** reviewing, writing. **Sandrine Faivre:** reviewing. **Muriel Hourseau:** data collection. **Béatrix Barry:** supervision, reviewing. **Esteban Brenet:** supervision, reviewing. **Diane Evrard:** original idea, writing.

## Funding

The authors have nothing to report.

## Disclosure

The authors have nothing to report.

## Ethics Statement

Authorisation to conduct this study was obtained from the local Ethical Committee (No‐IRB 00006477).

## Conflicts of Interest

The authors declare no conflicts of interest.

## Data Availability

The data that support the findings of this study are available from the corresponding author upon reasonable request.
